# Probable sarcopenia is associated with cognitive impairment among community-dwelling older adults: results from the FIBRA study

**DOI:** 10.1590/0004-282X-ANP-2020-0186

**Published:** 2021-05-01

**Authors:** Gabriela Cabett Cipolli, Ivan Aprahamian, Flávia Silva Arbex Borim, Deusivania Vieira Silva Falcão, Meire Cachioni, Ruth Calderia de Melo, Samila Sathler Tavares Batistoni, Anita Liberaleso Neri, Mônica Sanches Yassuda

**Affiliations:** 1 Universidade de São Paulo Escola de Artes Ciências e Humanidades São Paulo SP Brazil Universidade de São Paulo, Escola de Artes, Ciências e Humanidades, São Paulo SP, Brazil.; 2 Faculdade de Medicina de Jundiaí Departamento de Clínica Médica Jundiaí SP Brazil Faculdade de Medicina de Jundiaí, Departamento de Clínica Médica, Grupo de Investigação sobre Multimorbidade e Saúde Mental no Envelhecimento, Jundiaí SP, Brazil.; 3 Universidade Estadual de Campinas Programa de Pós-Graduação em Gerontologia Campinas SP Brazil Universidade Estadual de Campinas, Programa de Pós-Graduação em Gerontologia, Campinas SP, Brazil.; 4 Universidade de Brasília Faculdade de Ciências de Saúde Departamento de Saúde Coletiva Brasília DF Brazil Universidade de Brasília, Faculdade de Ciências de Saúde, Departamento de Saúde Coletiva, Brasília DF, Brazil.

**Keywords:** Cognitive Impairment, Cognition, Aged, Sarcopenia, Hand Strength, Comprometimento Cognitivo, Cognição, Idosos, Sarcopenia, Força da Mão

## Abstract

**Background:**

The link between sarcopenia and cognitive impairment has not yet been thoroughly evaluated, especially among older adults.

**Objective:**

To evaluate the relationship between probable sarcopenia and cognitive impairment among community-dwelling older adults in two Brazilian cities.

**Methods:**

Probable sarcopenia was assessed using the EWGSOP2 (2018) criteria. Thus, participants were classified as probably having sarcopenia if they had SARC-F (Strength, Assistance in walking, Rise from a chair, Climb stairs and Falls) ≥4 points and low grip strength. Cognitive function was evaluated through the Mini-Mental State Examination (MMSE), verbal fluency (VF) and clock drawing test (CDT).

**Results:**

In a sample of 529 older adults (mean age 80.8±4.9 years; mean education 4.2±3.67 years; 70.1% women), 27.3% of the participants had SARC-F≥4, 38.3% had low grip strength and 13.6% were classified as probable sarcopenia cases. After adjusting for possible confounders (age, sex, education, depression, diabetes, hypertension, leisure-time physical activity and obesity), probable sarcopenia was found to be associated with impairment in the MMSE (OR 2.52; 95%CI 1.42‒4.47; p=0.002) and in VF (OR 2.17; 95%CI 1.17‒4.01; p=0.014). Low grip strength was found to be associated with impairment in the MMSE (OR 1.83; 95%CI 1.18‒2.82; p=0.006) and in the CDT (OR 1.79; 95%CI 1.18‒2.73; p=0.006). SARC-F scores were found to be associated with impairment in the MMSE (OR 1.90; 95%CI 1.18‒3.06; p=0.008).

**Conclusion:**

The results suggested that probable sarcopenia and its components present a significant association with cognitive deficits among community-dwelling older adults. Future longitudinal studies will further explore the causal relationship.

## RESUMO

**Introdução:**

A ligação entre sarcopenia e comprometimento cognitivo ainda não foi completamente avaliada, especialmente entre os idosos.

**Objetivo:**

Avaliar a relação entre sarcopenia provável e comprometimento cognitivo entre idosos residentes na comunidade em duas cidades brasileiras.

**Métodos:**

A sarcopenia provável foi avaliada pelo critério EWGSOP2 (2018), portanto, os participantes foram classificados como tendo sarcopenia provável se tivessem SARC-F (em inglês, Strength, Assistance in walking, Rise from a chair, Climb stairs and Falls) ≥4 pontos e baixa força de preensão manual. A função cognitiva foi avaliada através do Miniexame do Estado Mental (MEEM), fluência verbal (FV) e teste do desenho do relógio (TDR).

**Resultados:**

Em uma amostra de 529 idosos (idade média 80,8±4,9 anos; escolaridade média 4,2 (±3,67) anos; 70,1% de mulheres), 27,3% dos participantes apresentaram SARC-F≥4, 38,3% apresentaram baixa força de preensão manual e 13,6% foram classificados com sarcopenia provável. Após o ajuste para possíveis fatores de confusão (idade, sexo, educação, depressão, diabetes, hipertensão, atividade física no lazer e obesidade), a sarcopenia provável foi associada ao comprometimento no MEEM (OR 2,52; IC95% 1,42‒4,47; p=0,002) e na FV (OR 2,17; IC95%=1,17‒4,01; p=0,014); a baixa força de preensão foi associada a comprometimento no MEEM (OR 1,83; IC95% 1,18‒2,82; p=0,006) e no TDR (OR 1,79; IC95% 1,18‒2,73; p=0,006); e os escores na SARC-F foram associados ao comprometimento no MEEM (OR 1,90; IC95% 1,18‒3,06; p=0,008).

**Conclusão:**

Os resultados sugerem que existe associação significativa entre sarcopenia provável e seus componentes com déficits cognitivos em idosos da comunidade. Futuros estudos longitudinais explorarão a relação causal.

Sarcopenia is a condition characterized by progressive and generalized loss of skeletal muscle mass and strength^1^. Several mechanisms are involved in the development and progression of sarcopenia^2^, and it has been associated with major adverse outcomes such as physical disability, falls, loss of quality of life and death^2^. In the current literature, variable prevalence is reported, ranging from 6 to 22% and depending on the population, setting and definition adopted^3^. In the early 2000s, the estimated costs attributed to sarcopenia reached US$ 18.5 billion in the United States^4^.

The first European Working Group on Sarcopenia in Older People (EWGSOP) recommended that the diagnosis of sarcopenia should be based on the presence of low muscle mass in association with low muscle strength or low physical performance^2^. According to the EWGSOP, low muscle mass should ideally be evaluated using dual-energy x-ray absorptiometry (DXA), muscle strength using a manual hand dynamometer and physical performance using gait speed. However, the Sarcopenia Definition and Outcomes Consortium stated that a) absolute or body mass index-adjusted grip strength is an important predictor of adverse health outcomes such as falls, instrumental activities of daily life disability and mortality; b) lean mass, as measured using DXA, is not a good marker of mobility impairment^5^.

In an updated EWGSOP consensus (EWGSOP 2), it was recommended that sarcopenia should be defined as a disease (muscular insufficiency) based on the use of the SARC-F (a brief screening instrument with five self-report questions about S=strength, A=ambulation, R=rise from a chair, C=climbing stairs and F=falling) and it should be diagnosed through the presence of low muscle strength, low muscle quality and/or low performance^3^. If all these criteria were present, the individual could be said to present severe sarcopenia^6^.

More recently, a few studies have demonstrated an association between sarcopenia and cognitive impairment^7^^,^^8^. Dementia is a predominantly geriatric syndrome that is considered to be the fifth leading cause of death worldwide. Its estimated prevalence among people age 60 years and over has been found to range from 4.7% in central Europe to 8.5% in Latin America and 8.7% in North Africa and the Middle East^9^. These numbers are expected to nearly double every 20 years, reaching 75 million by 2030 and 131.5 million by 2050^8^. About 58% of the people with dementia live in low and middle- income countries, but by 2050 this will rise to 68%^10^.

Six cross-sectional studies showed a significant relationship between sarcopenia and cognition, with participants’ mean age ranging from 69.9 to 82.3 years^11^^,^^12^^,^^13^^,^^14^^,^^15^^,^^16^. A longitudinal study observed that older adults with sarcopenia were at increased risk of cognitive decline after one year of follow-up, with mean ages of 73.9 years in the control group and 77.3 years in the group with sarcopenia^17^. However, the association between sarcopenia and cognition is still debatable, given that other three cross-sectional studies did not find any significant relationship, with mean ages ranging from 70.7 to 80.51 years^18^^,^^19^^,^^20^. Recently, a report from the ELSA-Brazil study showed that sarcopenia was associated with poorer performance in verbal fluency, and that low muscle strength was associated with poorer performance in all cognitive tests, in a sample of middle-aged and older adults with a mean age of 62.5 years^21^.

It is noteworthy that most of the studies exploring the association between sarcopenia and cognition have been conducted in high-income countries, mainly in Asia. These studies used DXA or bioimpedance to estimate muscle mass. However, the latter examinations may not be easily available in low and middle-income countries, either in primary care or in research settings. In addition, there is lack of data originating from Latin America. At the moment, there is only one previous study regarding the association between sarcopenia and cognitive performance that was conducted in a Latin American middle-income country^21^.

Szlejf et al.^21^ adopted the Foundation for the National Institutes of Health (FNIH) criteria for sarcopenia identification, using bioimpedance to evaluate muscle mass and a dynamometer to assess handgrip strength. Cognition was assessed using the CERAD word list, semantic verbal fluency and the trail-making test. The participants were 55 years and over. In the present study, we adopted the EWGSOP2 criteria for probable sarcopenia when the SARC-F was applied, and handgrip strength was assessed using a dynamometer. Cognition was assessed through fast easy-to-apply cognitive screening instruments, such as the Mini-Mental State Examination (MMSE), semantic verbal fluency (VF) and the clock drawing test (CDT), and the participants were 73 years and older. Differently from Szlejf et al.^21^, we aimed to assess whether a relationship between probable sarcopenia and cognition could be observed in an older sample, in which these conditions would be more prevalent, but, most importantly, using assessment strategies that would be easily available in most settings (SARC-F, a dynamometer and cognitive screening tests). Such a strategy might prove to be cost-effective and practical for identifying older adults at risk of these two potentially incapacitating conditions among older adults.

Therefore, the aims of the present study were:

- To examine whether there were any significant differences in common cognitive screening tests when older adults with and without probable sarcopenia were compared.
- To examine whether probable sarcopenia would be associated with cognitive deficits in screening tests of overall cognition (MMSE) and executive function (VF and CDT), in a sample of community-dwelling older adults in two locations in the state of São Paulo, Brazil.

## METHODS

### Participants and study design

A cross-sectional analysis was carried out among older adults over 73 years of age who were living in Ermelino Matarazzo, a subdistrict of the city of São Paulo, and in Campinas, both in the state of São Paulo, Brazil, in the years 2016‒17. These participants were recruited from a probabilistic sample of 1,284 community-dwelling older adults who had participated in the baseline measurements of the FIBRA study between 2008 and 2009. Detailed information about the baseline sample recruitment strategy, sample characteristics and main results are found elsewhere^22^. The aim of the FIBRA study was to estimate the prevalence of frailty and identify associated factors among older adults in probabilistic samples in seven Brazilian cities.

**Figure 1:**
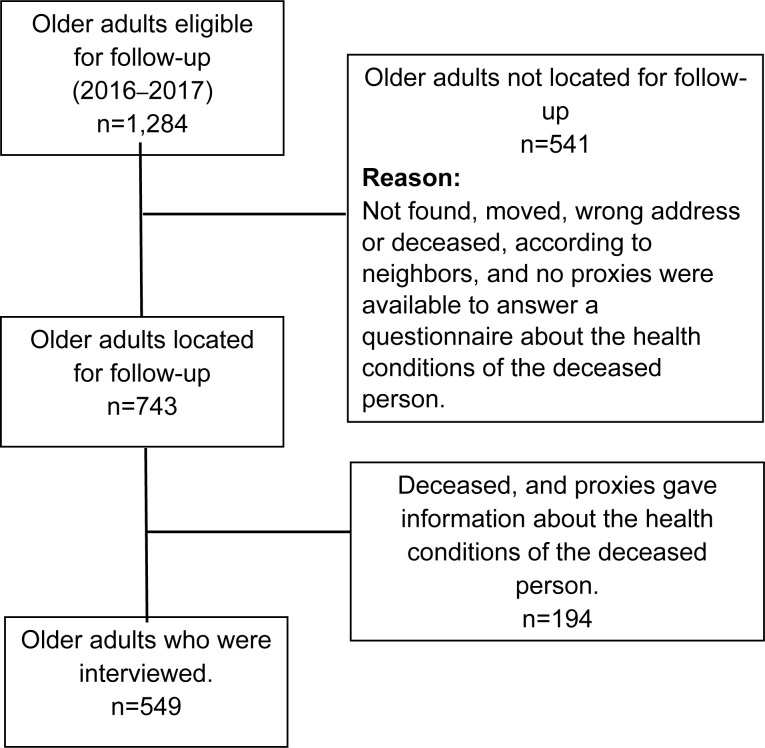
Follow-up sample flowchart. FIBRA study (2016‒2017).

Recruitment for the present study was carried out by visiting the participants’ homes. During these visits, two previously trained interviewers invited the participants to complete a follow-up protocol. The inclusion criteria for the follow-up assessment were: a) having participated in the first data collection for the FIBRA study in 2008‒09; b) absence of physical, language or noticeable cognitive impairment that could prevent participation; and c) presence of a family member, caregiver or close friend at the time of the interview. Out of the original 1,284 older adults, 549 were located and interviewed at home, 192 had died and 543 either were not found or refused to participate, as shown in Figure 1.

Data were collected between 2016 and 2017 in a single session, with an average duration of one hour. All participants completed a cognitive screening protocol, anthropometric measurements and measurements to assess the frailty phenotype (fatigue, weight loss, reduced gait speed, reduced grip strength and reduced activity level). Their body mass index was calculated.

Among the 549 older adults who composed the present sample, 130 participants presented a deficit in the MMSE. Based on Brucki et al.^23^, the cutoff scores for the MMSE were adjusted for years of schooling: 17 points for individuals who were illiterate and without formal education; 22 for those with 1 to 4 years of schooling; 24 for those with 5 to 8 years; and 26 for those with 9 years or more. For these participants with deficits, questions about health and functional status, observed cognitive status and mood were answered by a family member, caregiver or friend who knew the person well.

The 419 older adults who scored above the cutoffs in the MMSE answered the complete protocol, which included questions about self-reported health, functional status and psychosocial variables.

All the procedures were conducted in accordance with ethical principles relating to research on human beings, as stipulated by the Helsinki Convention and by the National Health Council of Brazil. All individuals who showed an interest in participating signed an informed consent statement. The project was approved under CAAE 49987615.3.0000.5404 and 92684517.5.1001.5404.

### Data collection

All the interviewers were undergraduate and postgraduate students in gerontology at the School of Arts, Sciences and Humanities of the University of São Paulo (EACH-USP) and at the State University of Campinas (UNICAMP) and underwent two months of training in the study procedures and protocols.

### Sarcopenia screening

Probable sarcopenia was identified using the strategy suggested by EWGSOP 2^6^^,^ in which the criteria were SARC-F≥4 points and presence of low handgrip strength.

SARC-F is a screening instrument based on five self-report questions about strength, ambulation, getting up from a chair, climbing stairs and falling. SARC-F scores range from 0 to 10 points^24^. For this study, the cutoff score for sarcopenia was four points^25^.

Handgrip strength in kilograms was assessed using a JAMAR® handheld dynamometer. The participants were asked to sit and bend their elbow and forearm at 90 degrees. They were then asked to hold the device using as much strength as possible. The size of the grip was adjustable, so that each participant, regardless of hand size, could feel comfortable while gripping the dynamometer. The test was performed three times with the dominant hand, with a one-minute rest between tests, and the simple mean from the three attempts was calculated. Following the EWGSOP2 criteria, the cutoff points for identifying low strength were taken to be <27 kg for men and <16 kg for women^26^.

### Demographic information

The demographic information investigated included age, gender and education level.

### Cognitive function

Cognitive performance was assessed using the MMSE, animal category VF and the CDT.

The MMSE briefly assesses spatial and temporal orientation, episodic memory, working memory (mental calculations), language and constructive praxis. The scores can range from zero to 30 points, such that higher values signal better performance^23^.

In the animal category VF, the participants are asked to name as many animals as possible within 60 seconds. This assesses the ability of the individual to retrieve information within a given semantic constraint. The education-adjusted cutoff scores were 9 animals for illiterate adults, 12 animals for those with 1 to 7 years of schooling and 13 animals for those with 8 or more years^27^.

The CDT assesses various cognitive dimensions, such as semantic memory, constructive praxis and executive functions regarding design planning. The scoring rules of Shulman et al.^28^ were used, ranging from 0 to 5 points, such that drawings with scores below 4 would be considered impaired^29^.

### Chronic diseases and physical activity

The number of chronic diseases was self-reported by the participants in a question which included a list of frequent diseases, such as hypertension, diabetes, depression and obesity.

Physical activity was measured using an adapted version of the Minnesota Leisure Activity Questionnaire^30^^,^^31^^,^^32^, which presents 18 dichotomous and structured response items for older adults. For each activity, the participants had to identify whether it was performed and, if so, inform the average number of times per month, considering the last year, and the average time, in minutes, spent on each occasion. Those who accumulated at least 120 minutes per week in vigorous physical activity and sports (>6 MET) or those who accumulated more than 150 minutes per week in physical exercise and moderate-intensity active sports (from >3 MET to <6 MET) were classified as active^30^^,^^31^^,^^32^.

### Statistical analyses

The chi-square test and Fisher's exact test were used to compare categorical variables. Logistic regression analyses were used to study the association between probable sarcopenia and cognitive impairment in the MMSE, VF and CDT. For each cognitive measurement, three models were tested (crude analysis, adjusted model 1 and adjusted model 2). The association between probable sarcopenia and cognitive impairment was firstly examined in the crude analysis. In adjusted model 1, this association was examined after adjusting for age, sex and education. In adjusted model 2, this association was examined after adjusting for age, sex, education, depression, diabetes, hypertension, physical activity and obesity. Similar models were run to examine the associations between SARC-F scores and cognitive impairment and between handgrip strength and cognitive impairment, so as to assess the individual component associations. All the statistical tests were two-tailed, and p-values at or below 0.05 were taken to be significant. The analyses were performed using the Stata software, version 15.0 (Stata Corp., College Station, USA).

## RESULTS

The participants’ mean age was 80.8±4.9 years, their mean education level was 4.2±3.67 years and 384 (70.1%) were women. Out of the total sample (n=529), 27.3% of the participants had SARC-F≥4, 38.3% had low grip strength and 13.6% were classified as having probable sarcopenia. The sociodemographic and cognitive characteristics are shown in Table 1 for the total sample and according to the probable sarcopenia classification. Participants with probable sarcopenia were significantly older, and, among them, there was a higher percentage of older adults with reduced physical activity and cognitive impairment, according to the MMSE, VF and CDT. Also, cognitive scores were significantly lower for participants with probable sarcopenia.

**Table 1:** Characteristics of the sample according to the presence of probable sarcopenia (n=529). FIBRA study (2016‒2017).

		Without probable sarcopenia n=457 (86.4%)	With probable sarcopenia n=72 (13.6%)	Total	p-value
Age, n (%)					
	73‒79	206 (45.0%)	19 (26.4%)	225 (42.5%)	0.003^a^
	˃80	251 (55%)	53 (73.6%)	304 (57.4%)	<0.001^b^
	M (SD)	80.2 (4.6)	83.0 (5.3)	80.8 (4.9)	
Sex, n (%) (women)		318 (85.5%)	54 (14.5%)	372 (70.1%)	0.337^a^
Education, n (%)					
	Illiterate	59 (13.5%)	11 (15.7%)	70 (13.8%)	
	1–4 years	263 (60.3%)	45 (64.3%)	308 (60.8%)	0.437^a^
	5–8 years	60 (13.8%)	10 (14.3%)	70 (13.8%)	0.047^b^
	9 years or more	54 (12.4%)	4 (5.7%)	58 (11.4%)	
	M (SD)	4.4 (3.8)	3.4 (2.9)	4.2 (3.6)	
BMI, n (%)					
	Eutrophic	196 (43.2%)	27 (38.0%)	223 (42.5%)	
	Low weight	89 (19.6%)	17 (24.0%)	106 (20.2%)	0.617^a^
	Overweight/obesity	169 (37.2%)	27 (38.0%)	196 (37.3%)	0.713^b^
	M (SD)	27.1 (4.9)	26.9 (6.1)	27.1 (5.1)	
Hypertension, n (%)					
	Yes	302 (68.1%)	54 (78.2%)	356 (69.5%)	0.090^a^
	No	141 (31.9%)	15 (21.8%)	156 (30.5%)	
Diabetes, n (%)					
	Yes	125 (28.3%)	46 (66.7%)	148 (29.0%)	0.396^a^
	No	316 (71.6%)	23 (33.3%)	362 (71.0%)	
Depression, n (%)					
	Yes	71 (16.1%)	9 (13.0%)	80 (15.7%)	0.512^a^
	No	369 (83.9%)	60 (87.0%)	429 (84.3%)	
Physical activity, n (%)					
	Active	212 (46.5%)	19 (26.4%)	231 (43.8%)	<0.001^a^
	Inactive	244 (53.5%)	53 (73.6%)	297 (56.2%)	
SARC-F, n (%)					
	0–3 points	385 (84.0%)	0 (0.0%)	385 (72.6%)	<0.001^a^
	≥4 points	73 (16.0%)	72 (100%)	145 (27.4%)	<0.001^b^
	M (SD)	1.9 (1.93)	5.9 (1.6)	2.4 (2.3)	
Handgrip strength, n (%)					
	Normal	332 (72.5%)	0 (0.0%)	332 (62.6%)	<0.001^a^
	Low strength	126 (27.5%)	72 (100%)	198 (37.4%)	<0.001^b^
	M (SD)	23.1 (10.7)	14.2 (4.4)	21.7 (10.5)	
MMSE, n (%)					
	Without deficit	359 (80.0 %)	44 (61.1%)	403 (77.3%)	<0.001^a^
	With deficit	90 (20.0%)	28 (38.9%)	118 (22.6%)	<0.001^b^
	M (SD)	23.7 (4.0)	21.2 (5.3)	23.1 (4.6)	
Verbal fluency, n (%)					
	Without deficit	201 (46.1%)	19 (27.1%)	220 (43.5%)	0.003^a^
	With deficit	235 (53.9%)	51 (72.9%)	286 (56.5%)	0.005^b^
	M (SD)	10.7 (4.3)	9.2 (4.2)	10.4 (4.3)	
Clock drawing test, n (%)					
	Without deficit	179 (39.1%)	18 (25.0%)	197 (37.2%)	0.021^a^
	With deficit	278 (60.9%)	54 (75.0%)	332 (62.8%)	0.005^b^
	M (SD)	2.4 (1.8)	1.9 (1.8)	2.4 (1.8)	

M: mean; SD: standard deviation; MMSE: Mini-Mental State Examination; BMI: body mass index; p-value<0.05 shows statistical significance;

a Pearson's chi-square test;

b Student's t-test.

Table 2 shows the associations of probable sarcopenia, low handgrip strength and SARC-F with cognitive impairment. In the adjusted models (model 1 and model 2), probable sarcopenia was associated with impairment in the MMSE and VF. SARC-F scores were associated with impairment in the MMSE. Low handgrip strength was associated with impairment in the MMSE and CDT, and marginally with VF.

**Table 2:** Association of probable sarcopenia and its defining components with cognitive performance (n=529). FIBRA study (2016‒2017).

		Overall cognition (MMSE)		Verbal fluency test (VF)		Clock drawing test (CDT)	
		OR (95%CI)	P	OR (95%CI)	P	OR (95%CI)	P
Probable sarcopenia							
	Crude analysis	2.53 (1.49‒4.30)	0.001	2.29 (1.31‒4.01)	0.004	0.21 (0.08‒0.50)	0.159
	Model 1^a^	2.57 (1.49‒4.42)	0.001	2.21 (1.24‒3.92)	0.007	2.02 (1.11‒3.66)	0.021
	Model 2^b^	2.52 (1.42‒4.47)	0.002	2.17 (1.17‒4.01)	0.014	1.64 (0.90‒3.04)	0.111
SARC-F							
	Crude analysis	1.83 (1.18‒2.83)	0.007	1.32 (0.88‒1.97)	0.167	1.28 (0.86‒1.92)	0.215
	Model 1^a^	1.88 (1.20‒2.85)	0.005	1.37 (0.90‒2.06)	0.134	1.20 (0.78‒1.83)	0.399
	Model 2^b^	1.90 (1.18‒3.06)	0.008	1.22 (0.78‒1.90)	0.370	0.97 (0.62‒1.53)	0.929
Low handgrip strength							
	Crude analysis	1.89 (1.27‒2.81)	0.002	1.51 (1.05‒2.16)	0.025	1.79 (1.23‒2.60)	0.002
	Model 1^a^	1.95 (1.29‒2.95)	0.001	1.45 (0.99 ‒2.11)	0.052	1.89 (1.27‒2.82)	0.002
	Model 2^b^	1.83 (1.18‒2.82)	0.006	1.48 (0. 99‒2.21)	0.052	1.79 (1.18‒2.73)	0.006

MMSE: Mini-Mental State Examination; VF: verbal fluency; CDT: clock drawing test; OR: *Odds* Ratio; 95%CI: 95% confidence interval;

a Model 1: logistic regression adjusted for age, sex and education;

b Model 2: logistic regression adjusted for age, sex, education, depression, diabetes, hypertension, leisure-time physical activity and obesity.

## DISCUSSION

The present study explored the relationship between cognition (evaluated using three screening tools: MMSE, VF and CDT) and probable sarcopenia, as measured through SARC-F≥4 and low grip strength among community-dwelling older adults. Most of the participants were women and presented low educational background. Among the participants with probable sarcopenia, there was higher frequency of cognitive impairment, and their scores in the MMSE, VF and CDT were significantly lower. In the adjusted regression analyses (model 1 and model 2), probable sarcopenia was associated with impairment in the MMSE and VF. Regarding individual components, SARC-F scores were significantly associated with impairment in the MMSE and handgrip strength with impairment in the MMSE and the CDT, and there was a trend in relation to VF.

In a recent systematic review and meta-analysis, it was observed that sarcopenia was significantly associated with cognitive impairment (pooled OR 2.50; 95%CI 1.26‒4.92; p=0.008)^33^. Thus, the present study is in agreement with previous investigations and it adds evidence that this association is observed among older community-dwelling adults living in middle-income countries. It is noteworthy and new that this association was also evident when the recently proposed criteria for probable sarcopenia were used.

Recently, a study in Brazil^21^ investigated the association between cognitive performance (as assessed using the CERAD Word List, VF and Trail-Making Test) and sarcopenia, as defined by the FNIH criteria. That study was cross-sectional with 5,038 participants from the ELSA-Brazil study, aged≥55 years. After adjustment for possible confounders, sarcopenia and low muscle mass were found to be associated with lower performance in VF. Low muscle strength was found to be associated with poorer performance in all three tests.

The present study used SARC-F and handgrip strength to identify probable sarcopenia and similar associations were observed. Here, probable sarcopenia was found to be associated with impairment in overall cognition and VF. In both studies, it could be seen that these associations seemed to be driven by handgrip strength, which had the highest correlations with the cognitive measurements. In the present analyses, the MMSE was the cognitive screening tool that showed the most significant correlation with probable sarcopenia and its individual components. Taken together, both studies suggest that the sarcopenia-cognition association is not restricted to specific cognitive domains.

A narrative review reported that handgrip strength measurement were associated with cognitive decline, regardless of age and presence of comorbidities^34^. These findings indicated that the association of sarcopenia and cognitive function was probably motivated by changes in muscle strength. Future longitudinal studies are needed to clarify this possibly causal association. Most previous studies that addressed the relationship between sarcopenia and cognitive performance were cross-sectional, as so was ours, with one exception. In a longitudinal study, Nishiguchi et al.^17^ evaluated cognition and sarcopenia among 131 Japanese seniors (121 non-sarcopenic and 10 sarcopenic) at baseline and after 12 months. After this period, those with sarcopenia showed greater cognitive decline.

The biological processes underlying the association between sarcopenia and cognitive impairment are unclear, but there are several plausible explanations. First, cognitive impairment may lead to less physical activity and poor dietary intake, which could lead to excessive muscle loss in seniors^35^. Second, chronic low-grade age-related inflammation, characterized by elevated interleukin-6 and tumor necrosis factor-α, for example, has also been reported as an important causal factor for both sarcopenia and lower cognitive performance^36^. Third, excessive oxidative stress related to chronic diseases, including neurodegenerative ones, can also cause loss of skeletal muscle mass, thus causing sarcopenia^37^.

Frailty has also been previously associated with cognitive impairment in many studies^38^. Currently, the relationship between frailty and sarcopenia is under discussion, considering that these conditions are related to similar negative health outcomes and have shared pathophysiology^38^. Sarcopenia and frailty are risk factors that can co-occur in a single individual^39^. Some overlap between sarcopenia and frailty is expected, since muscle function (handgrip strength and gait speed) is included in the definitions of sarcopenia and in the physical frailty phenotype proposed by Fried et al.^40^. Also, weight loss is a criterion for frailty and contributes to sarcopenia^39^. However, apart from muscle function, the definitions of frailty include other components that are more indirectly related to the musculoskeletal system^39^. Sarcopenia is often considered to be a precursor syndrome or the physical component of frailty^38^.

The limitations of our study need to be addressed. We acknowledge that the sample size may have caused some error of inference or may have reduced the power of the analyses. Also, underestimation of the number of participants with probable sarcopenia may have occurred, given that the participants were independent older adults who were survivors from the baseline sample. On the other hand, among the strengths of the study, we can cite that participants were community-dwelling adults who had been randomly selected for the original study, thus minimizing selection bias.

In conclusion, our study is the first to correlate probable sarcopenia (EWGSOP2 criteria), assessed using SARC-F plus handgrip strength, with performance in cognitive screening tests. We found that probable sarcopenia was significantly associated with cognitive deficits in this sample. These results have important implications for geriatric care, such as highlighting the importance of assessing cognitive impairment among older adults when sarcopenia is present. Longitudinal studies are needed to explore causal associations between sarcopenia and cognitive impairment.
